# Comparative Analysis of Fecal Microbiota of Grazing Mongolian Cattle from Different Regions in Inner Mongolia, China

**DOI:** 10.3390/ani11071938

**Published:** 2021-06-29

**Authors:** Han Aricha, Huasai Simujide, Chunjie Wang, Jian Zhang, Wenting Lv, Xirnud Jimisi, Bo Liu, Hao Chen, Chen Zhang, Lina He, Yinxue Cui, Ruijuan Gao, Chen Aorigele

**Affiliations:** 1 College of Animal Science, Inner Mongolia Agricultural University, Hohhot 010018, China; 15248146527@163.com (H.A.); smjd_2010@163.com (H.S.); ZhangJian280613@163.com (J.Z.); drhan_mgl@126.com (X.J.); bsm_2021@126.com (B.L.); bsm_mgl@163.com (H.C.); arh_han@126.com (C.Z.); na15374712742@163.com (L.H.); baism_2010@163.com (Y.C.); 2 College of Veterinary Medicine, Inner Mongolia Agricultural University, Hohhot 010018, China; chunjiewang200@soho.com (C.W.); ccmm0508@yeah.net (W.L.); han_aruhan@163.com (R.G.)

**Keywords:** Mongolian cattle, gut microbiota, microbial diversity, geography, 16S rRNA sequencing, high throughput sequencing

## Abstract

**Simple Summary:**

Recently, there has been increasing attention focused on the intestinal microflorae of animals due to their critical role in maintaining health and preventing disease. With the improvement of the Chinese national economy and the people’s material standard of living, the beef cattle industry is growing rapidly to meet the growing market demand for beef. Mongolian cattle is a precious genetic resource in China and an excellent cattle breed in Inner Mongolia. However, updated research on topics concerning the gut microbiota of Mongolian cattle are absent. Therefore, this study focused on the differences in the gut microbiota composition of Mongolian cattle in different geographical environments. The gut microbiota composition of the Mongolian cattle from the grasslands was relatively similar, while that from the desert areas was different. The results of this study contribute to our understanding of the influence of geographical factors on the composition of gut microbiota in Mongolian cattle.

**Abstract:**

Mongolian cattle from China have strong adaptability and disease resistance. We aimed to compare the gut microbiota community structure and diversity in grazing Mongolian cattle from different regions in Inner Mongolia and to elucidate the influence of geographical factors on the intestinal microbial community structure. We used high throughput 16S rRNA sequencing to analyze the fecal microbial community and diversity in samples from 60 grazing Mongolian cattle from Hulunbuir Grassland, Xilingol Grassland, and Alxa Desert. A total of 2,720,545 high-quality reads and sequences that were 1,117,505,301 bp long were obtained. Alpha diversity among the three groups showed that the gut microbial diversity in Mongolian cattle in the grasslands was significantly higher than that in the desert. The dominant phyla were *Firmicutes* and *Bacteroidetes*, whereas *Verrucomicrobia* presented the highest abundance in the gut of cattle in the Alxa Desert. The gut bacterial communities in cattle from the grasslands versus the Alxa Desert were distinctive, and those from the grasslands were closely clustered. Community composition analysis revealed significant differences in species diversity and richness. Overall, the composition of the gut microbiota in Mongolian cattle is affected by geographical factors. Gut microbiota may play important roles in the geographical adaptations of Mongolian cattle.

## 1. Introduction

Gut microbiota plays a crucial role in maintaining host health and has important functions, such as participation in metabolism, supplying of nutrients and energy to the host [1], and inhibition of growth of pathogenic micro-organisms [2]. Previous studies have suggested that the gut microbiota is affected by several factors, such as host genome [3], diet [4], and geography [5,6,7]. Recent studies have compared the fecal microbiota communities in cattle and Bactrian camels and found that *Firmicutes* is the predominant phylum. Hierarchical clustered heatmap analysis revealed that the microbial community composition within the Bactrian camel groups was relatively similar but distinct from that in the cattle [8]. Recently, some studies have focused on the relationship between the community structure of gut microbiota and geography. Song et al. (2017) [6] compared the gut microbiota of Chinese black bears from different provinces and suggested that the gut microbiota can be affected by geography. A vast number of studies investigating the role of the intestinal microbiota of humans from different areas have been published [9,10,11]. However, few studies have assessed the geographical relationship of gut microbiomes in cattle.

Mongolian cattle are dual-purpose cattle and an excellent cattle breed in Inner Mongolia, China. This species is characterized by its resistance to diseases, environmental adaptation, and crude feed resistance. The Xilingol (XM) Grassland, Hulunbuir (HM) Grassland, and Alxa (AM) Desert are the main production areas of Mongolian cattle. This study aimed to compare the community structure and composition of the gut microbiota in Mongolian cattle from different regions and reveal the influence of geographical factors and dietary habits on the intestinal microflora to provide scientific support for future research and the healthy breeding of Mongolian cattle.

## 2. Materials and Methods

### 2.1. Sample Collection

All animal studies were conducted according to the guidelines established by the Institutional Animal Care and Use Committee of the Inner Mongolian Agricultural University (Hohhot, China). Procedures were performed under the national standard Guidelines for Ethical Review of Animal Welfare (GB/T 35892-2018).

There were 20 fecal samples collected from healthy grazing Mongolian cattle (3- to 4-year-old females) in XM, HM, and AM, respectively. The cattle were grazed naturally and drank water freely. They were not dewormed with any product before sampling. Fecal samples were obtained in sterile tubes immediately after the natural defecation of the animals and were transported to the laboratory using liquid nitrogen, and stored at −80 °C until DNA extraction.

### 2.2. Genome DNA Extraction

The total genomic DNA from fecal samples was extracted using the hexadecyl trimethyl ammonium bromide (CTAB) method [12]. Agarose gel (1%) electrophoresis was used to determine the concentration and purify the extracted DNA. The extracted DNA was diluted to 1 ng/μL with sterile water.

### 2.3. PCR Amplification and Hiseq Sequencing

Using the diluted genomic DNA as a template, the v3-v4 hypervariable region of the 16S rRNA gene was amplified using specific primers 338F (5′-ACTCCTACGGGAGGCAGCA-3′) and 806R (5′-GACTACHVGGGTWTCTAAT-3′). All polymerase chain reactions (PCR) were carried out with 30 μL of reaction mixture with 15 μL of Phusion^®^ High-Fidelity PCR Master Mix (New England Biolabs, Ipswich, MA, USA), 0.2 μM of forward and reverse primers, and approximately 10 ng of template DNA. Thermal cycling consisted of initial denaturation at 98 °C for 1 min, followed by 30 cycles of denaturation at 98 °C for 10 s, annealing at 50 °C for 30 s, elongation at 72 °C for 30 s, and a final extension at 72 °C for 5 min. The PCR products were mixed in equal density ratios. Then, 2% agarose gel electrophoresis was performed to detect the PCR products. The samples with the intensity of the main band between 400 to 450 bp were selected for further experiments. PCR products were then purified using the Gene JETTM Gel Extraction Kit (Thermo Scientific, Waltham, MA, USA). Library quality was assessed using the Qubit@ 2.0 Fluorometer (Thermo Scientific). After Qubit and q-PCR quantification, a library was constructed and sequenced on the Illumina Hiseq 2500 platform.

### 2.4. Sequence Analysis

According to the Quantitative Insights into Microbial Ecology (QIIME) quality control process, the high-quality clean tags were obtained through specific filtration of the raw tags [13,14]. Sequence analysis was performed using the Uparse software [15]. Sequences with ≥97% similarity were assigned to the same operational taxonomic unit (OTU). A representative sequence for each OTU was filtered for further annotation. OTUs were taxonomically analyzed by the Ribosomal Database Project (RDP) Classifier algorithm [16].

### 2.5. Data Statistics

Subsequent analyses of alpha diversity and beta diversity were based on normalized data on OTU abundance information that used the sequence number criteria corresponding to the sample with the least sequences [17]. The complexity of species diversity was analyzed using the alpha diversity indices, namely Chao1 and ACE (community richness), Shannon and Simpson (diversity), and Good’s coverage (sequencing depth), which were calculated using Mothur [18]. The difference in species complexity was evaluated using beta diversity, and the weighted UniFrac was calculated using QIIME. Principal coordinate analysis (PCoA) was performed before clustering analysis, and principal coordinates were obtained from complex multidimensional data and visualized. Phylogenetic trees were constructed using the unweighted pair-group method with arithmetic means (UPGMA) clustering, and the distance matrix was interpreted and combined with the bacterial community histogram to evaluate microbiome similarity and taxonomic differences among the different groups or samples. A Venn diagram was implemented using the R package to show unique and shared OTUs. We used linear discriminant analysis (LDA) with LEfSe [19], an algorithm biomarker discovery that identifies taxa characterizing the differences among three metadata classes, and the default setting LDA score filter value was 4. High LDA scores reflect significantly a higher abundance of certain taxa. Tukey’s test was used to analyze whether species diversity was significant between groups. *p* < 0.05 was considered statistically significant. Sequencing services, database construction, and statistical analysis were performed by Beijing Novogene Technology Co. Ltd. (Beijing, China).

## 3. Results

### 3.1. 16S rRNA Gene Sequencing

**Figure 1:**
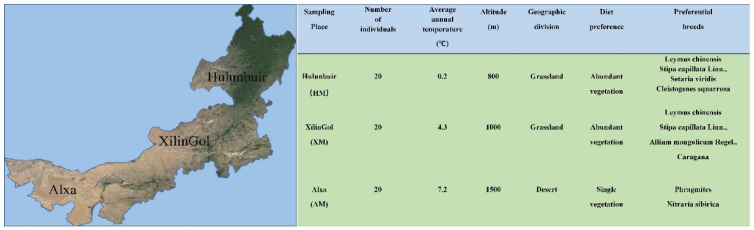
The detailed characteristics of the Hulunbuir (HM), Xilingol (XM), and Alxa (AM) regions of Inner Mongolia, China.

The detailed characteristics of the three study groups are shown in Figure 1. A total of 5,423,215 raw reads were generated from the 60 fecal samples through Illumina Hiseq sequencing. After filtering out low-quality reads, 2,950,033 clean reads with an average sequence length of 410.3 bp were retained from 1,117,505,301 base pairs of sequences. Finally, 2,720,545 base pairs of high-quality reads were obtained from all samples for further analysis.

### 3.2. Microbial Diversity

All reads were classified into 2650 operational taxonomic units (OTUs) (Table S1), which belonged to 165 families and affiliated to 24 phyla. Good’s coverage estimations of all samples were between 98.7% and 99.2%, which indicated that the sequencing depth was sufficient to detect the microbial community in fecal samples. To better understand OTU diversity in each group, we compared the alpha diversity among Mongolian cattle from the different regions according to the Chao 1, ACE, and the Simpson and Shannon indices, which were used to estimate bacterial community richness and diversity, respectively. As shown in Table 1, the observed species decreased in the following order, HM >, XM >, and AM. Microbial communities in the HM and the XM groups displayed significantly higher richness and diversity than in the AM group (*p* < 0.001). This indicated that the HM and the XM groups have a high bacterial diversity.

### 3.3. Beta Diversity

**Figure 2:**
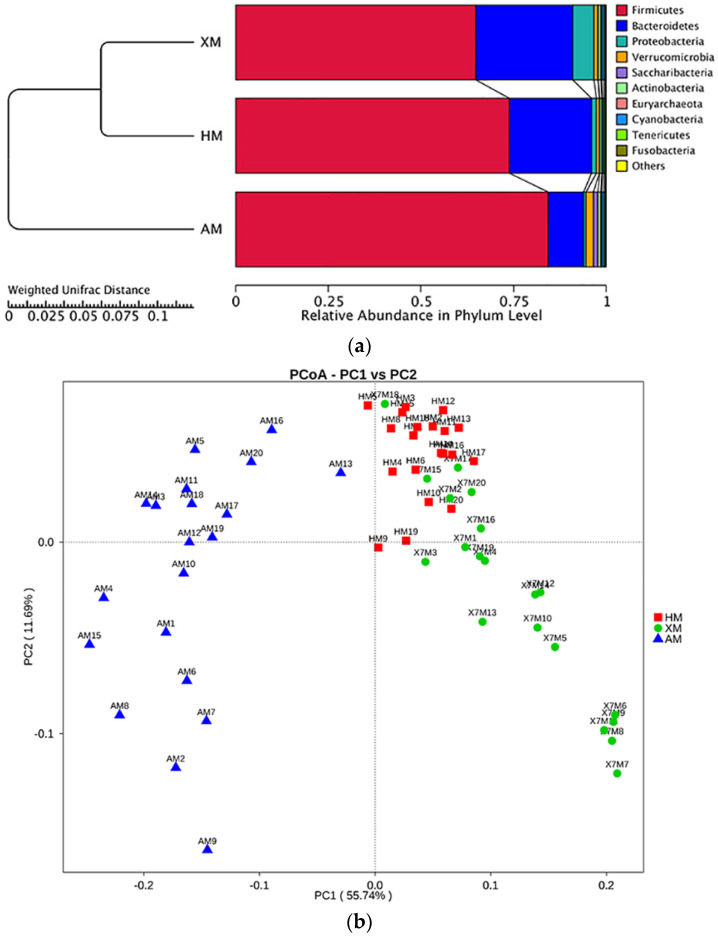
Relationship of the gut microbiota of the Mongolian cattle from three different regions. (**a**) Clustering analysis of the evolution of the gut microbiotas in the Mongolian cattle from Xilingol (XM), Hulunbuir (HM), and Alxa (AM). Gut microbiota trees were generated by using the UPGMA algorithm based on the Unweighted Unifrac distances generated by QIIME software. (**b**) PcoA based on Weighted Unifrac distance of Mongolian cattle microbial community in three different regions.

The differences in the diversity of the gut microbiota among the different groups were further compared using the UPGMA clustering tree based on the weighted UniFrac distances. Interestingly, as shown in Figure 2a, the gut microbiota in the HM and the XM groups were clustered together, but those Mongolian cattle in the AM group were excluded. The PCoA was used to examine the community structure of the gut microbiota. The weighted UniFrac distances were calculated based on a phylogenetic tree. In the PCoA plot, each symbol represents one fecal sample (Figure 2b). Similar to the result of the UPGMA clustering tree analysis mentioned above, the bacterial communities in the HM and the XM groups clustered closely and separated from that of the AM group along principal coordinate axis 1 (PC1), which explained the highest proportion of variation (55.74%).

### 3.4. Bacterial Community Taxonomic Composition

**Figure 3:**
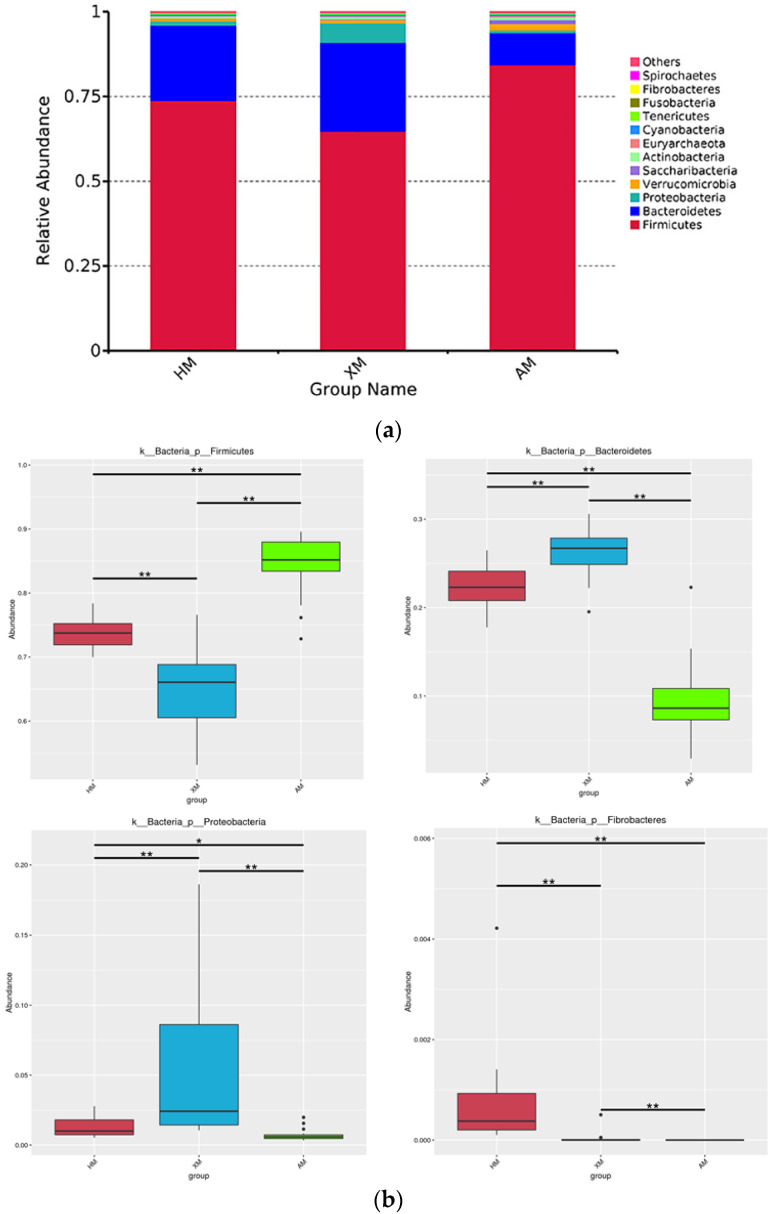
Taxonomic profiles at the phyla. (**a**) Relative abundance of bacterial groups from the HM, XM, and AM groups at the phylum level. (**b**) At the phyla level, the statistical chart of species significance difference of 60 Mongolian cattle fecal samples from HM, XM, and AM groups.

All sequences obtained from the three groups were classified to the phyla and family levels using the Mothur program. Twenty-four bacterial phyla were identified. The top 12 abundant phyla with the highest abundances were selected (Figure 3a). *Firmicutes* was the dominant flora represented by 64.77 ± 6.72%–84.36 ± 4.66% of the 16S rRNA gene sequences, followed by *Bacteroidetes* with 9.44 ± 4.33%–26.19 ± 2.66%. The proportion of the same bacteria in the different groups varied. The abundance of *Firmicutes* and *Verrucomicrobia* (84.36 ± 4.66% and 1.99 ± 2.44%) in the AM group was higher than that in the HM and the XM groups (73.77 ± 2.54% and 64.77 ± 6.72%, and 0.81 ± 0.32% and 1.00 ± 0.99%, respectively), while the abundance of *Bacteroidetes* (9.44 ± 4.33%) was lower than that in the other groups (22.22 ± 2.44% and 26.19 ± 2.66%). The XM group presented a higher abundance of *Proteobacteria* (5.70 ± 5.85%) in the feces than the other groups (1.31 ± 0.71% and 0.71 ± 0.41%) (Table S2). Interestingly, *Fibrobacteres* was not found in the AM group (0%), but the highest abundance was in the HM group. At the phylum level, the abundance distribution box of the different species was plotted (Figure 3b). *Firmicutes*, *Bacteroidetes*, *Proteobacteria,* and *Fibrobacteres* differed significantly among the three groups (*p* < 0.001).

**Figure 4:**
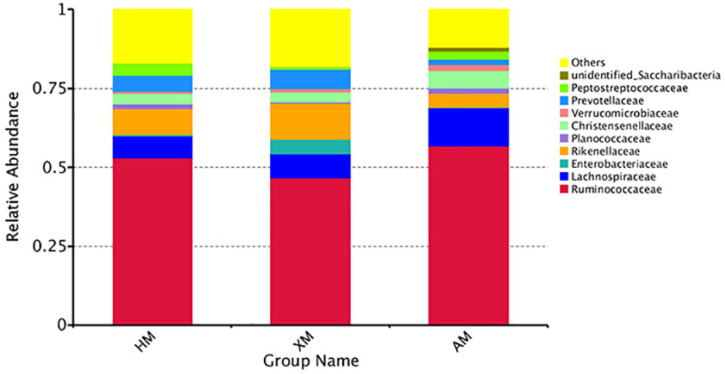
Relative abundance of bacterial groups from the HM, XM, and AM groups at the family level.

At the family level, 165 families were detected in all samples. The top ten abundant families with the highest abundance at the family level were selected (Figure 4). *Ruminococcaceae* was the predominant family in all of the groups represented by 56.75±5.91% of the 16S rRNA gene sequences. The second most abundant family was *Lachnospiraceae* (12.09 ± 5.79%). In the AM group, the proportions of *Ruminococcaceae*, *Lachnospiraceae*, *Christensenellaceae*, and *Verrucomicrobiaceae* were (56.75 ± 5.91%, 12.09 ± 5.79%, 5.57 ± 2.22%, and 1.99 ± 2.14%) higher than that in the HM and the XM groups (52.91 ± 2.69%, 7.13 ± 1.45%, 3.20 ± 0.36%, and 0.79 ± 0.32%; and 46.72 ± 6.78%, 7.66 ± 1.29%, 3.20 ± 0.82%, and 0.99 ± 0.99%, respectively). The relative abundances of *Enterobacteriaceae* and *Rikenellaceae* in the XM group were 4.58 ± 6.06% and 11.30 ± 1.49%, which was higher than that in the HM and the AM groups (0.36 ± 0.59% and 8.31 ± 1.22%; and 0.24 ± 0.27% and 4.31 ± 3.57%, respectively). The abundance of *Peptostreptococcaceae* was low (0.80 ± 0.37%). At the family level, the abundance of unclassified bacteria in the present study was from 12.02% to17.96% (Table S3).

### 3.5. Microbial Signatures

**Figure 5:**
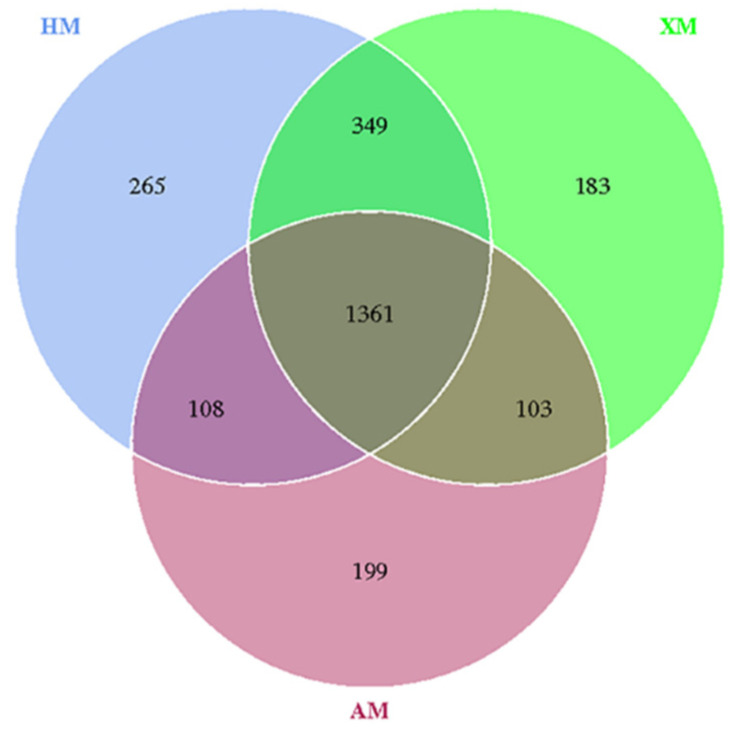
The Venn diagram based on OTUs of Mongolian cattle fecal microbiota from the three different regions.

**Figure 6:**
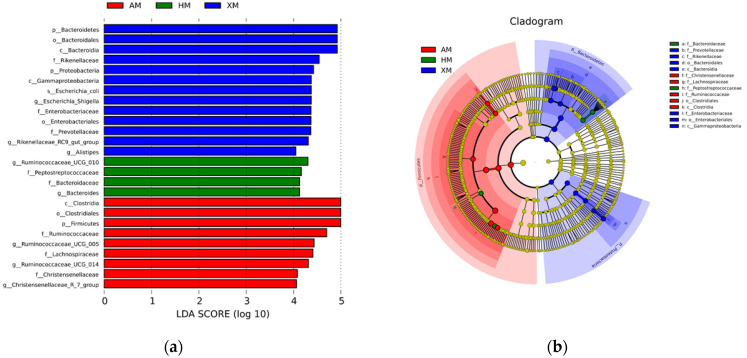
LDA Effect Size (LEfSe) algorithm was used on OTU tables to determine taxa that best characterize each biological class. (**a**) Cladogram showing the phylogenetic distribution of the bacterial lineages between Mongolian cattle from the three different regions. Different colored regions represent different constituents (blue, XM; green, HM; red, AM). (**b**) Circles from the inside out indicate phylogenetic levels from genus to phylum.

There were 1361 OTUs found in all 60 fecal samples (Figure 5), of which 265 OTUs were unique for the HM group, 183 for the XM group, and 199 for the AM group. The LEfSe analysis was used to determine the taxa that most likely explained the differences among the Mongolian cattle from the three different regions. The result of the LEfSe analysis confirmed the significant enrichment of the phylum *Bacteroidetes*, order *Bacteroidales*, and class *Bacteroidia* in the XM group. The genus *Ruminococcaceae_UCG-010* and family *Peptostreptococcaceae* were enriched in the HM group, and the class *Clostridia*, order *Clostridiales*, phyla *Firmicutes*, and family *Ruminococcaceae* were enriched in the AM group (Figure 6a,b).

## 4. Discussion

In the present study, we evaluated the fecal microbiota of 60 grazing Mongolian cattle from three different regions. This technology helped us gain a deeper understanding of the bacterial diversity in fecal samples based on the identification of the core gut flora in grazing Mongolian cattle.

Recently, there has been increasing attention on animal intestinal microflora because of its critical role in maintaining health and preventing disease, therefore, determining the environmental factors that shape the gut microbiota were the focus of this study. There are several reports on the differences in the microbiome structure of the intestinal tract of humans from different geographical environments with different diets and genetic backgrounds [20,21,22]. However, there have been few reports on the relationship between environmental factors and gut microbiome profiles of grazing cattle.

Different geographical environments can change the diet and living habits of a host, thus affecting the composition of the gut microbiome. In the present study, a high diversity of gut microbes were found in the cattle from the Xilingol Grasslands and the Hulunbuir Grasslands. In comparison, the diversity of the gut microbiome in the cattle from the Alxa Desert was low. The result may be because there are the various forages, such as *Leymus chinensis*, *Stipa capillata* Linn., *Medicago sativa*, *Allium mongolicum* Regel., and *Caragana* edible for the cattle in the Xilingol and Hulunbuir grasslands, whereas there are only herbaceous species like *Phragmites*, *Nitraria sibirica*, and *Kalidium gracile* obtainable for the cattle in the Alxa Desert. This point can be supported by the report from Lau et al. [23]. They compared the fecal microbiome of Hong Kong’s omnivorous cattle and traditional cattle in southern China and found that microbiota diversity increased with diet variation.

The UPGMA cluster analysis showed that the fecal microbiota in Mongolian cattle in both the Hulunbuir and Xilingol grasslands clustered together while that in the Alxa Desert separated. This clustering may depend on the environment of the host, as it is a stronger determinant of the microbial community structure [8]. PCoA with weighted UniFrac distance revealed that the intestinal microflora of Mongolian cattle was related to the environment, and the grassland areas overlapped considerably, however, the desert areas were clustered independently. The Xilingol and Hulunbuir grasslands have a continental climate, with an average annual rainfall of 180–280 mm, and abundant native vegetation. In contrast, the Alxa Desert has arid areas with drought and limited rain. The average annual rainfall is 88–180 mm and vegetation is sparse. The substantial difference in diet and environment in the different regions may lead to the variations in the gut microbial communities in the cattle.

At the phylum level, the core gut microbiome in the present study consisted of *Firmicutes* and *Bacteroidetes*. The two phyla have been shown to constitute the majority of gut-associated phylotypes in a variety of mammalian species [24,25,26,27], suggesting that *Firmicutes*, and *Bacteroidetes* in particular, played a beneficial role in the microbial ecology of the gut of mammals, including cattle. *Firmicutes* was the most abundant in the AM group samples. Since the cattle consume mainly a single vegetation species in the desert areas, the cattle need to consume large amounts of forage for energy, and additionally, they need to spend energy to maintain their body temperature. Furthermore, the microbial community with a high abundance of *Firmicutes* in their gut allows the cattle to absorb energy from food as much as possible to maintain their body functions. *Fibrobacteres* are considered major degraders of plant biomass in the gut of herbivores [28]. They degrade cellulose enabling animals to absorb it. The cattle in the Hulunbuir Grassland feed on fiber-rich herbs, while in the Alxa Desert they always feed on a single species of herb. Interestingly, *Verrucomicrobia* was more abundant in the gut of the cattle from the AM group. A previous study found that *Verrucomicrobia* became more abundant in the human gut after the use of broad-spectrum antibiotics [29]. This microbe type, which increased in abundance after the introduction of antibiotics in the human gut, was present in large quantities in the intestinal tract of the grazing Mongolian cattle in the Alxa Desert for a long time. We speculate that the existence of *Verrucomicrobia* was related to the extremely strong disease resistance of Mongolian cattle. At the family level, the top three relatively abundant core intestinal microbial families were *Ruminococcaceae*, *Lachnospiraceae*, and *Rikenellaceae*. Several members of *Ruminococcaceae* were the common inhabitants in the gut and feces of several mammals, including cattle [30,31]. It is surprising to have found that cattle in the Alxa Desert had an abundant population of *Lachnospiraceae*. *Lachnospiraceae* is related to the production of short-chain fatty acids. It stimulates the production of butyric acid in the intestinal tract, providing the energy for the growth of intestinal epithelial cells, and also has an anti-tumor effect [32,33]. *Lachnospiraceae* acts as a barrier in the gut and has been found in a reduced abundance in children with Crohn’s Disease [34]. The rich *Lachnospiraceae* in the gut of grazing Mongolian cattle in the Alxa Desert protects the gut and acts as a barrier, helping the Mongolian cattle to adapt to the harsh living environment and reduce the incidence of intestinal diseases.

The bacterial communities in the samples from the two grasslands (Hulunbuir (HM) and Xilingol (XM)) were significantly different from those from the desert (Alxa (AM)). The Xilingol and Hulunbuir grasslands have similar geography, climatic conditions, and vegetation. The Alxa Desert is far away from the two grasslands, and with an arid climate. Although we found different fecal microbiota compositions among the Xilingol and Hulunbuir grasslands, there were no significant differences in species richness and community diversity. The samples from grasslands presented significantly higher community diversity and species richness than those of the samples from the Alxa Desert. The results may suggest that the gut microbiota pattern in the cattle could be affected by geographical factors.

## 5. Conclusions

In conclusion, the intestinal flora in the cattle from the Hulunbuir and Xilingol grasslands were clustered more closely than those from the Alxa Desert. Our data further revealed that the existence of *Verrucomicrobia* in the gut of the Mongolian cattle in the Alxa Desert can be related to the strong disease resistance of the cattle. Our research promotes our understanding of the effect of geography on species diversity of gut microbiota in their host. However, further studies using, for instance, metagenomics or an increased sample size, are still needed for a comprehensive understanding of the diversity of the gut microbiome community in cattle.

## Figures and Tables

**Table 1:** Alpha diversity index of grazing Mongolian cattle from different regions.

Group	Observed_Species	Community Diversity		Community Richness		
		Shannon	Simpson	Chao1	Ace	Goods_Coverage (%)
HM	1144.300 ± 50.761 ^a^	8.001 ± 0.113 ^a^	0.985 ± 0.002 ^a^	1368.287 ± 92.585 ^a^	1359.763 ± 78.557 ^a^	98.70 ± 0.001 ^b^
XM	1096.950 ± 50.217 ^b^	7.884 ± 0.296 ^a^	0.982 ± 0.008 ^a^	1301.873 ± 71.984 ^b^	1302.415 ± 70.688 ^b^	98.70 ± 0.001 ^b^
AM	823.900 ± 70.532 ^c^	7.326 ± 0.324 ^b^	0.980 ± 0.006 ^b^	951.889 ± 197.518 ^c^	938.770 ± 100.786 ^c^	99.20 ± 0.001 ^a^
*p*-Value	<0.0001	<0.0001	0.03	<0.0001	<0.0001	<0.0001

Note: Data in the table were mean ± standard error. Different shoulder letters indicate significant difference (*p* < 0.05), while the same letters indicate insignificant difference (*p* > 0.05).

## Data Availability

All the raw data during the current study are available from the corresponding author.

## Supplementary Materials

The following are available online at https://www.mdpi.com/article/10.3390/ani11071938/s1, Table S1: OTU table of the 60 samples, including the OTU species and sequence number contained in each sample, as well as species annotation information for each OTU. Table S2: The relative abundance of 60 samples at the phylum level. Table S3: The relative abundance of 60 samples at the family level.
